# Association Analysis of Nuclear Receptor Rev-erb Alpha Gene (*NR1D1*) and Japanese Methamphetamine Dependence

**DOI:** 10.2174/157015911795017065

**Published:** 2011-03

**Authors:** Taro Kishi, Tsuyoshi Kitajima, Kunihiro Kawashima, Tomo Okochi, Yoshio Yamanouchi, Yoko Kinoshita, Hiroshi Ujike, Toshiya Inada, Mitsuhiko Yamada, Naohisa Uchimura, Ichiro Sora, Masaomi Iyo, Norio Ozaki, Nakao Iwata

**Affiliations:** 1 Department of Psychiatry, Fujita Health University School of Medicine, Toyoake, Aichi 470-1192, Japan; 2 Japanese Genetics Initiative for Drug Abuse, Japan; 3 Department of Neuropsychiatry, Okayama University Graduate School of Medicine, Dentistry and Pharmaceutical Sciences, Okayama 700-8558, Japan; 4 Department of Psychiatry, Seiwa Hospital, Institute of Neuropsychiatry, Tokyo 162-0851, Japan; 5 National Institute of Mental Health, National Center of Neurology and Psychiatry, Ichikawa 272-0827, Japan; 6 Department of Neuropsychiatry, Kurume University School of Medicine, Kurume 820-0011, Japan; 7 Department of Psychobiology, Department of Neuroscience, Tohoku University Graduate School of Medicine, Sendai 980-8576, Japan; 8 Department of Psychiatry, Chiba University Graduate School of Medicine, Chiba 260-8677, Japan; 9 Department of Psychiatry, Nagoya University Graduate School of Medicine, Nagoya 466-8850, Japan

**Keywords:** Orphan nuclear receptor Rev-erb alpha gene (*NR1D1*), methamphetamine dependence, tagging SNPs, linkage disequilibrium.

## Abstract

Several investigations suggested abnormalities in circadian rhythms are related to the pathophysiology of psychiatric disorders, including drug addiction. Recently, orphan nuclear receptor rev-erb alpha and glycogen synthase kinase-3 β (GSK-3β) were shown to be important circadian components. In addition, the orphan nuclear receptor rev-erb alpha is a key negative feedback regulator of the circadian clock. These evidences indicate that rev-erb alpha gene (NR1D1) is a good candidate gene for the pathogenesis of methamphetamine dependence. To evaluate the association between NR1D1 and methamphetamine dependence, we conducted a case-control study of Japanese samples (215 methamphetamine dependence and 232 controls) with three tagging SNPs selected by HapMap database. Written informed consent was obtained from each subject. This study was approved by the ethics committees at Fujita Health University, Nagoya University Graduate School of Medicine and each participating member of the Institute of the Japanese Genetics Initiative for Drug Abuse (JGIDA). We did not detect an association between NR1D1 and Japanese methamphetamine dependence patients in allele/genotype-wise analysis, or the haplotype analysis. Our findings suggest that NR1D1 does not play a major role in the pathophysiology of methamphetamine dependence in the Japanese population.

## 1 INTRODUCTION

Several investigation suggest that abnormalities in circadian rhythms may be involved in the pathophysiology of psychiatric disorders, including drug addiction [1-3]. The evidence for this relation is discussed in more detail in our previous papers and a review by Barnard and Nolan [4-7].

Recently, Mohawk and colleagues reported that mice which were arrhythmic due to a lack of circadian clock genes showed circadian locomotor rhythms when treated with methamphetamine [8]. If patients with drug addiction disorders had disrupted circadian rhythms, it is possible that taking methamphetamine helps to restore circadian rhythms of taking into methamphetamine to keep to circadian rhythm. Several animal studies have shown that methamphetamine increased expression of circadian clock molecule genes such as *Per1*, *Per2*, *Bmal1*, and *Npas2 *in the brain [9-11]. Recently, we detected the significant association between prokineticin 2 receptor gene (*PROKR2*), which is one of the clock genes, and Japanese methamphetamine dependence patients [12].

Recently, rev-erb alpha and glycogen synthase kinase-3 β (GSK-3β), which are known to be important circadian components [13], have been discussed in relation with lithium. Several studies have revealed that lithium affects circadian rhythms through GSK-3β [14-16]. Yin *et al.* showed that Rev-erb alpha is a target of GSK-3β kinase activity, needed to mediate this effect to regulate circadian rhythms [13]. These evidences indicate that rev-erb alpha gene (*NR1D1*) is a good candidate gene for the pathogenesis of methamphetamine dependence.

To evaluate the association between *NR1D1* and methamphetamine dependence, we conducted a case-control study of Japanese samples (215 methamphetamine dependence and 232 controls) with three tagging SNPs selected by HapMap database.

## 2 MATERIALS AND METHODS

### 2.1 Subjects

The subjects in the association analysis were 215 METH deprendence patients (175 males and 40 females; mean age ± standard deviation (SD) 36.3 ± 11.4 years) and 232 healthy controls (187 males and 45 females; 36.4 ± 11.3 years). The age and sex of the control subjects did not differ from those of the methamphetamine dependence patients. All subjects were unrelated to each other, ethnically Japanese, and lived in the central area of Japan. The patients were diagnosed according to DSM-IV criteria with consensus of at least two experienced psychiatrists on the basis of unstructured interviews and a review of medical records. One hundred ninety-seven of the subjects with METH dependence had a diagnosis of co-morbid METH induced psychosis. METH-induced psychosis patients were divided into two categories of psychosis prognosis, the transient type and the prolonged type, which showed remission of psychotic symptoms within 1 month and after more than 1 month, respectively, after the discontinuance of methamphetamine consumption and beginning of treatment with neuroleptics; 112 patients (56.9%) were the transient type, and 85 patients (42.1%) were the prolonged type. One hundred eighty-two subjects with METH-induced psychosis also had dependence on drugs other than METH. Cannabinoids were the most frequency abused drugs (21.4%), followed by cocaine (9.09%), LSD (9.09%), opioids (7.69%), and hypnotics (7.69%). Subjects with METH-induced psychosis were excluded if they had a clinical diagnosis of psychotic disorder, mood disorder, anxiety disorder or eating disorder. More detailed characterizations of these subjects have been published elsewhere [17, 18]. All healthy controls were also psychiatrically screened based on unstructured interviews. None had severe medical complications such as liver cirrhosis, renal failure, heart failure or other Axis-I disorders according to DSM-IV.

The study was described to subjects and written informed consent was obtained from each. This study was approved by the Ethics Committee at Fujita Health University, Nagoya University School of Medicine and and each participating member of the Institute of the Japanese Genetics Initiative for Drug Abuse (JGIDA).

### 2.2 SNPs Selection and Linkage Disequilibrium (LD) Evaluation

We first consulted the HapMap database (release#20/phase II, Jan 2006, www.hapmap.org, population: Japanese Tokyo, minor allele frequencies (MAFs) of more than 0.05) and included 5 SNPs covering *NR1D1* (5’-flanking regions including about 750 bp from the initial exon and about 1 kb downstream (3’) from the last exon: HapMap database contig number chr17: 35501880..35510616). Then three ‘tagging (tag) SNPs’ were selected with the criteria of r^2^ threshold greater than 0.8 in ‘pair-wise tagging only’ mode using the ‘Tagger’ program (Paul de Bakker, http://www/broad.mit.edu/mpg/tagger), in Haploview [19], three ‘tag SNPs’ (rs939347, rs2071427 and rs3744805) were selected for the following association analysis.

### 2.3 SNPs Genotyping

We used TaqMan assays (ABI: Applied Biosystems, Inc., Foster City, CA,) for all SNPs. One allelic probe was labeled with FAM dye and the other with the fluorescent VIC dye. The plates were heated for 2 min at 50°C and 95°C for 10 min, followed by 45 cycles of 95°C for 15 s and 58°C for 1 min. Please refer to ABI for the primer sequence. Detailed information is available on request.

### 2.4 Statistical Analysis

Genotype deviation from the Hardy-Weinberg equilibrium (HWE) was evaluated by chi-square test (SAS/Genetics, release 8.2, SAS Japan Inc, Tokyo, Japan).

Marker-trait association analysis was used to evaluate allele- and genotype-wise association with the chi-square test (SAS/Genetics, release 8.2, SAS Japan Inc, Tokyo, Japan), and haplotype-wise association analysis was conducted with a likelihood ratio test using the COCAPHASE2.402 program [20]. We used the permutation test option as provided in the haplotype-wise analysis to avoid spurious results and correct for multiple testing. Permutation test correction was performed using 1000 iterations (random permutations). Power calculation was performed using a genetic power calculator [21]. The significance level for all statistical tests was 0.05. 

## 3 RESULTS

The LD structure of *NR1D1* from the HapMap database samples can be seen our previous paper [22]. Genotype frequencies of all SNPs were in HWE. We did not detect between all tagging SNPs and METH dependence in the Japanese population in the allele /genotype-wise (Table **1**) or haplotype-wise analysis (*P* _haplotype_= 0.775). In addition, we found no association between four tagging SNPs and METH-induced psychosis patients in the allele /genotype-wise (Table **1**) or haplotype-wise analysis (*P* _haplotype_= 0.699).

In the power analysis, we obtained more than 80% power for the detection of association when we set the genotype relative risk at 1.63-1.86 in METH dependence for *NR1D1 *under a multiplicative model of inheritance 

## 4 DISCUSSION

We did not find an association between *NR1D1* and Japanese METH dependence and METH-induced psychosis patients. Therefore, we reasoned that *NR1D1* may not play an important role in the pathophysiology of METH dependence and METH-induced psychosis in the Japanese population. However, because our samples are small, we considered that there is a possibility of statistical error in these results.

Recently, we reported that *NR1D1* was not associated with major depressive disorder and selective serotonin reuptake inhibitor response to major depressive disorder [7, 22]. On the other hand, we detected the associations between *PROKR2* and not only mood disorders including major depressive disorder and bipolar disorder but also METH dependence and METH-induced psychosis in the Japanese population [12, 23]. We hypothesized that mood disorders and drug addiction have common susceptibility genes.

A few points of caution should be mentioned with respect to our results. Firstly, the lack of association may be due to small sample size. Ideal samples for this study are METH use disorder samples with and without dependence and psychosis. Because we had only a few METH use disorder samples without dependence and psychosis, and we wanted to avoid statistical error, we did not perform an association analysis with these samples. Secondly, we did not include a mutation scan to detect rare variants. We designed the study based on the common disease-common variants hypothesis [24]. Further investigation will be required, such as medical resequencing using larger samples. However, statistical power is needed to evaluate the association of rare variants. To overcome these limitations, a replication study using larger samples or samples of other populations will be required for conclusive results [25].

In conclusion, our results suggest that *NR1D1* may not play a role in the pathophysiology of METH dependence in the Japanese population. However, because we did not perform a mutation scan of* NR1D1*, a replication study using a larger sample may be required for conclusive results.

## Figures and Tables

**Table 1:** Association Analysis of *NRIDI* with Methamphetamine Dependence and Methamphetamine-Induced Psychosis

SNP ID^a^	Phenotype^b^	MAF^c^	N	Genotype Distribution^d^			P-Value^e^		
				M/M	M/m	m/m	HWE	Genotype	Allele
rs939347	Control	0.483	232	59	121	52	0.502		
A>G	METH dependence	0.498	215	55	106	232	0.838	0.770	0.703
	METH-induced psychosis	0.487	197	51	100	46	0.824	0.956	0.944
rs2071427	Control	0.469	232	66	114	52	0.835		
G>A	METH dependence	0.458	215	65	103	47	0.470	0.918	0.726
	METH-induced psychosis	0.452	197	59	98	40	0.952	0.856	0.597
rs3744805	Control	0.489	232	62	113	57	0.699		
A>G	METH dependence	0.516	215	51	106	58	0.850	0.720	0.419
	METH-induced psychosis	0.518	197	49	92	56	0.363	0.659	0.405

a major allele>minor allele

b METH: methamphetamin

c MAF: minor allele frequency

d M: major allele, m: minor allele

e Hardy-Weinberg equilibrium
